# Xylem-Inspired Hydrous Manganese Dioxide/Aluminum Oxide/Polyethersulfone Mixed Matrix Membrane for Oily Wastewater Treatment

**DOI:** 10.3390/membranes12090860

**Published:** 2022-09-05

**Authors:** Teng Sam Yun, Pei Ching Oh, Moau Jian Toh, Yun Kee Yap, Qin Yi Te

**Affiliations:** 1 Department of Chemical Engineering, Universiti Teknologi PETRONAS, Seri Iskandar 32610, Perak, Malaysia; 2 CO_2_ Research Centre (CO2RES), R&D Building, Universiti Teknologi PETRONAS, Seri Iskandar 32610, Perak, Malaysia

**Keywords:** ultrafiltration, oily wastewater, superhydrophilic mixed matrix membrane, hydrous manganese dioxide, aluminum oxide

## Abstract

Ultrafiltration membrane has been widely used for oily wastewater treatment application attributed to its cost-efficiency, ease of operation, and high separation performance. To achieve high membrane flux, the pores of the membrane need to be wetted, which can be attained by using hydrophilic membrane. Nevertheless, conventional hydrophilic membrane suffered from inhomogeneous dispersion of nanofillers, causing a bottleneck in the membrane flux performance. This called for the need to enhance the dispersion of nanofillers within the polymeric matrix. In this work, in-house-fabricated hydrous manganese dioxide–aluminum oxide (HMO-Al_2_O_3_) was added into polyethersulfone (PES) dope solution to enhance the membrane flux through a xylem-inspired water transport mechanism on capillary action aided by cohesion force. Binary fillers HMO-Al_2_O_3_ loading was optimized at 0.5:0.5 in achieving 169 nm membrane mean pore size. Membrane morphology confirmed the formation of macro-void in membrane structure, and this was probably caused by the hydrophilic nanofiller interfacial stress released in PES matrix during the phase inversion process. The superhydrophilic properties of PES 3 in achieving 0° water contact angle was supported by the energy-dispersive X-ray analysis, where it achieved high O element, Mn element, and Al elements of 39.68%, 0.94%, and 5.35%, respectively, indicating that the nanofillers were more homogeneously dispersed in PES matrix. The superhydrophilic property of PES 3 was further supported by high pure water flux at 245.95 L/m^2^.h.bar, which was 3428.70% higher than the pristine PES membrane, 197.1% higher than PES 1 incorporated with HMO nanofiller, and 854.00% higher than PES 5 incorporated with Al_2_O_3_ nanofillers. Moreover, the excellent membrane separation performance of PES 3 was achieved without compromising the oil rejection capability (98.27% rejection) with 12 g/L (12,000 ppm) oily wastewater.

## 1. Introduction

Tremendous growth in the petrochemical industries has been witnessed around the world throughout the past decades. The biggest challenge of the industry is to separate the micro-scale emulsified oil. Typically, the oil droplet sizes occur in stabilized emulsified oil droplet (<20µm), dispersed oil droplet (20–150 µm), and free-floating oil droplet (>150 µm) [1,2]. Conventional oily wastewater treatment methods rely on the floatation, coagulation, and biological treatment technology. The major drawbacks of these technologies are complexity, formation of secondary pollutants, and scum interaction on equipment [3]. Furthermore, these conventional methods incur high energy consumption, eventually leading to high operating cost [4]. On top of that, stringent environmental regulations that restrict the oil discharge limit to 10–20 ppm has raised awareness of membrane separation technology [5,6].

In recent decades, membrane separation is a promising technology in treating oily wastewater due to its high separation efficiency, low energy consumption, and lower complexity. Generally, ultrafiltration membrane with pore sizes ranging between 1 and 100 nm is considered to be optimum in producing high-quality permeate from oily wastewater treatment [7]. However, membranes submerged in high concentration oily wastewater are vulnerable to the oil molecules’ deposition and adsorption, potentially reducing the remaining useful life of the membrane. Numerous past research has been performed in enhancing the hydrophilicity of membrane surface through incorporating hydrophilic nanofillers into polymeric membrane to improve membrane flux and anti-fouling performance [8,9]. Although incorporating hydrophilic nanofillers into polymeric matrix is effective in enhancing membrane surface hydrophilicity [10,11], an excessive amount of the nanofillers will affect the membranes’ mechanical properties due to incompatibility between the nanofillers with the polymeric matrix, resulting in formation of pinholes [12].

Recently, Gohari et al. [11] found that incorporating hydrous manganese oxide (HMO) into the polyethersulfone polymer matrix was able to achieve high water flux and good organic solute rejection rate. In the meantime, Doraisammy et al. [13] reported that incorporating HMO nanoparticles into polyethersulfone (PES) polymeric matrix led to water flux of 32 L/m^2^.h.bar with 82% oil rejection efficiency. Furthermore, Lai et al. [10] introduced binary fillers comprising HMO and titanium dioxide (TiO_2_) into PES polymer matrix at various weight ratios. From their study, it was found that these binary fillers were able to achieve good water flux and oil rejection, with 100% improvement in water flux compared to the pristine PES membrane. Pang et al. [14] incorporated zinc oxide (ZnO) and multi-walled carbon nanotube (MWCNT) binary fillers into PES membrane, achieving 40 L/m^2^.h.bar with 88.51% rejection on 50 mg/L concentration of humic acid. Reports from Lai et al. and Pang et al. indicated that a synergistic effect took place, enhancing the membrane mean pore size. The release of the interfacial stress during the phase inversion process consequently leads to the formation of larger macropores, creating a pathway for water to penetrate through the membrane, thus increasing the water flux.

Several research studies on incorporating Al_2_O_3_ into polymeric matrix increased the hydrophilicity and flux of the MMMs [15,16,17]. Researchers found that a steric effect could enhance the dispersion of the fillers in the polymeric matrix [18,19]. For instance, the dissociative adsorption of the water molecules on the alumina particles (Al_2_O_3_) would lead to the formation of the Al-OH group due to the hydroxylation effect. Upon exposure to high temperature, the surface of the amphoteric oxide alumina experiences a dehydroxylation effect, forming the oxygen bridge site (Al-O-Al) [20]. Therefore, the steric effects of dual fillers would induce the repulsive forces, thus minimizing the aggregation of the single fillers caused by the strong Van der Waals’ forces, leading to homogeneous nanofiller distribution. An investigation by Mojtahedi et al. showed the addition of 0.5 wt % Al_2_O_3_ in PSF polymeric matrix enhanced the water flux to 80 L/m^2^.h.bar at 3 bar [15]. In addition, an optimization study that was carried out for Al_2_O_3_ in the PES polymeric matrix achieved high water flux of 252 L/m^2^.h.bar at 0.05 wt % under 1.03 bar operating pressure [16]. Furthermore, Razmgar et al. found that addition of 3 wt % of Al_2_O_3_ in PVDF/PVA matrix achieved the highest water flux at 32 L/m^2^.h.bar under the operating pressure of 5–6 bar [17]. Thus, incorporation of Al_2_O_3_ into the polymeric matrix could enhance membranes’ flux performance.

Meanwhile, HMO nanoparticles appear as small and poorly ordered particles (also known as core-corona microsphere polycrystalline structure) due to swift reaction between permanganate and manganate ions (i.e., swift nuclei formation) [21]. The continued growth of uniform nuclei within the corona dense core through the Ostwald ripening process would enhance the polymer chain interaction properties [22].

Recently, the incorporation of dual nanofillers into polymeric membranes was reported to introduce synergistic effects in enhancing the pore structure of membrane. The release of the interfacial stress during the phase inversion process consequently leads to the formation of larger macropores, creating a pathway for water to penetrate through the membrane, thus increasing the water flux. Although the reports incorporated Al_2_O_3_ or HMO nanofillers in various polymeric membranes, the nanofillers were individually evaluated. In this research work, we examined the potential of dual fillers HMO-Al_2_O_3_ on PES MMMs without the aid of pore opening agent, such as polyvinylpyrrolidone (PVP) and polyether glycol (PEG), in oily wastewater treatment application. Varying ratios of HMO and Al_2_O_3_ were used to determine the separation performance and intrinsic properties of the membranes in a high-concentration synthetic emulsified oily wastewater environment.

## 2. Materials and Methods

### 2.1. Materials

Commercial polyethersulfone (PES, nominal granule size 3 mm) in flake shape supplied by Sigma-Aldrich was used as the main polymer. *N*-Methyl-2-pyrrolidone (NMP, ≥99.0%) was used as a solvent to dissolve the PES polymer. Potassium permanganate (KMnO_4_, ≥99%) was supplied by Bendosen, whilst magnesium sulfate heptahydrate (MgSO_4_·7H_2_O) and sodium hydroxide (NaOH) were supplied by Merck Sdn. Bhd. Inorganic nanofillers aluminum dioxide (Al_2_O_3_) powder was supplied by Sigma-Aldrich. Crude oil was obtained from Terengganu oil terminal, and sodium dodecyl sulfate (SDS, ≥99%) supplied by Sigma-Aldrich was used as a surfactant for the preparation of synthetic emulsified oily wastewater. The core-corona hierarchical structure HMO was attributed to the high OH functional group distributed around the MgSO_4_, as depicted elsewhere [23]. All the chemicals received were used without further purification.

### 2.2. Preparation of Dope Solution

HMO nanoparticles were synthesized as reported by Parida by oxidizing the manganese ions using potassium permanganate with the ratio of KMnO_4_ to MgSO_4_·7H_2_O at 1:1.5 (*w*/*w*) [24]. Next, PES polymer (15 wt %, 1.17 mL) was dried in the vacuum oven at 65 °C for 10 h and stored in a desiccator until the next usage. Predetermined amounts of HMO/Al_2_O_3_ nanoparticles were initially dissolved into the NMP solvent (85 wt %, 8.65 mL) for efficient dispersion of nanoparticles. Then, the solution was sonicated for 30 min at 50 °C [10]. Dried PES polymer was added dropwise by priming technique to improve the compatibility of nanoparticles in the PES polymeric matrix. The dope solution was stirred for at least 24 h at 500 rpm and 70 °C until homogeneous [25]. The same method was applied for the pristine PES membrane dope solution preparation, without adding nanoparticles. Subsequently, the dope solutions were degassed for 30 min at room temperature and left standing overnight. The membranes were denoted as PES 0, PES 1, PES 2, PES 3, PES 4, and PES 5, wherein the membrane separation performance was evaluated at various HMO-Al_2_O_3_ loadings (i.e., 0:0, 1:0, 0.75:0, 0.5:0.5, 0.25:0.75, 0:1). 

### 2.3. UF Mixed Matrix Membrane (MMM) Preparation

The dope solution was evenly spread on a glass plate by using a BGD 206 film applicator (Biuged Laboratory Instruments Co Ltd., Guangzhou, China) with a 200 µm thickness gap. The casted film on the glass plate was immediately immersed into a deionized water coagulant bath for solvent and non-solvent exchange phase inversion. The membrane film was kept in deionized water for 24 h to remove the excess solvent. After that, the pristine membrane and MMMs were air dried for 24 h before we conducted the UF membrane performance evaluation. 

### 2.4. Membrane Separation Experiments

#### 2.4.1. Synthetic Oily Wastewater Preparation

The crude oil obtained from Terengganu crude oil terminal was used to prepare 12 g/L (12,000 ppm) synthetic oily wastewater. This was done by mixing with deionized water and sodium dodecyl sulfate (SDS) under vigorous stirring at 1000 rpm for 24 h until the formation of a uniform yellowish color. The ratio of crude oil to SDS was 9:1 (*w*/*w*) [26]. The crude oil preparation was conducted the day before the UF experiment by considering the oil coalescence as described by Gohari et al. [27]. The oil droplet sizes were characterized using Zetasizer Nano ZSP (Malvern Instrument Inc., Southborough, MA, USA). The refractive index of oil droplets and water (dispersant) were 1.5 and 1.333, respectively [26]. The average oil droplets size obtained for the synthetic oily wastewater was 291.9 nm.

#### 2.4.2. Ultrafiltration of Synthetic Oily Wastewater

**Figure 1:**
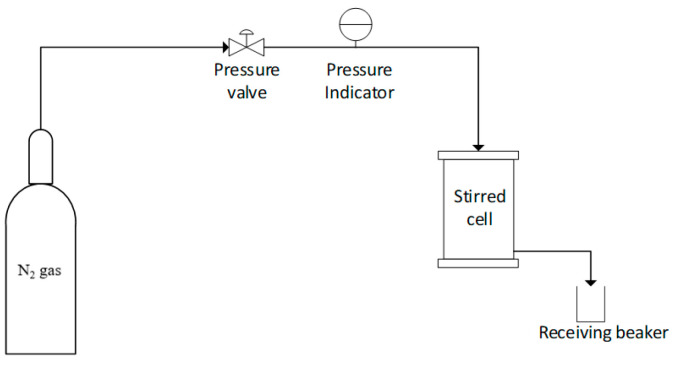
UF stirred cell test rig.

Dead-end filtration stirred cell with 28.7 cm^2^ membrane effective area was used to evaluate the pure water flux performance by applying 2 bar operating pressure for 30 min membrane compaction. After that, the operating pressure was reduced to 1 bar. The filtered permeate was collected for every 30 min in 1 h, and the average values were reported. Figure 1 shows the UF test rig. 

Pure water flux (*J_w_*_1_) was evaluated on the basis of Equation (1), where *Q_p_* is the volume of permeate collected, *A* is the membrane effective area (m^2^), and *t* is the operating hour (h).

(1) $$J_{w1}=\frac{Q_{p}}{A\times t}$$

The oil rejection efficiency was calculated using Equation (2) by determining the synthetic oil concentration in feed and permeate by using a UV–VIS spectrophotometer (Shimadzu UV mini-1240, Kyoto, Japan) at a wavelength of 308 nm. *C_p_* represents permeate concentration and *C_F_* represents synthetic oil feed concentration. Similar steps were repeated for PES 0 using 5 bar as the compaction pressure and 4 bar as the operating pressure, due to its high membrane resistance properties, induced by the hydrophobic nature of PES polymer.

(2) $$R=\left(1-\frac{C_{p}}{C_{f}}\right)\times 100\%$$

Upon the completion of oil rejection evaluation, the stirred cell was refilled with deionized water for 30 min, and the membranes were cleaned under the same operating conditions as pure water flux test for all the membranes. The pure water flux, *J_w2_*, was re-measured after deionized water flowed for 30 min following the oil rejection test. The flux recovery ratio was determined through Equation (3).

(3) $$FRR=(\frac{J_{W2}}{J_{W1}})\times 100\%$$

Next, the fouling resistance test, which included intrinsic membrane resistance (*R_m_*), irreversible membrane resistance (*R_ir_*), reversible membrane resistance (*R_r_*), and total resistance (*R_T_*) were evaluated on the basis of Equations (4)–(7) [11].

(4) $$R_{m}=(\frac{\Delta P}{\mu \times J_{W1}})\times 100\%$$

(5) $$R_{ir}=(\frac{\Delta P}{\mu \times J_{W2}})-R_{m}$$

(6) $$R_{r}=(\frac{\Delta P}{\mu \times J_{oil}})-R_{m}-R_{ir}$$

(7) $$R_{t}=R_{m}+R_{ir}+R_{r}$$

where *J_oil_* represents the oil flux (L/m^2^·h·bar), *μ* is 8.9 × 10^−4^ Pa·s, and ∆*P* represents the operating pressure.

### 2.5. Membrane Physicochemical Characterization

Membrane characterization was performed to identify the membrane morphology, porosity and pore size, hydrophilicity, functional group, and diffraction pattern.

#### 2.5.1. Membrane Morphology

The surface and cross-section morphology of pristine PES membrane and Al_2_O_3_/HMO incorporated MMMs were observed through scanning electron microscopy (SEM) (TM3030 HITACHI, Tokyo, Japan) at 15 kV accelerating voltage. A 1 k magnification was used for cross-section morphology, and 2 k magnification was applied for surface morphology. The membrane samples were dried in a vacuum oven overnight and cryogenically fractured using liquid nitrogen [14,28]. Furthermore, energy dispersion X-ray (EDX) was performed to analyze the elements on the membrane surface.

#### 2.5.2. Membrane Porosity and Pore Size Measurement

The dry weights of PES 0 to PES 5 membranes were measured and recorded. The membrane thicknesses were measured using a thickness gauge meter (Mitutoyo Absolute digital 547, Kawasaki, Japan). After that, the membranes were immersed in deionized water overnight, and the wet membrane weight was recorded. Thus, the membrane porosity was determined using Equation (8).

(8) $$\varepsilon \left(\%\right)=\left(\frac{W_{w}-W_{d}}{\rho \times A\times L}\right)\times 100\%$$

where *W_w_* represents the weight of wet membrane (g), *W_d_* is the weight of dry membrane (g), *ρ* is the water density (g/mm^3^), *A* is the membrane effective area (mm^2^), and *L* is the membrane thickness (mm). 

Next, the membrane pore size was estimated on the basis of the Guerout–Elford–Ferry equation in Equation (9) [10].

(9) $$r=\sqrt{\frac{\left(2.9-1.75\varepsilon \right)\times 8\eta lQ}{\varepsilon \times A\times \Delta P}}$$

where *ε* is the membrane porosity, *η* = 8.9 × 10^−4^ Pa.s represents water viscosity, *l* is the membrane thickness (m), *Q* is the volume of water collected per second (m^3^/s), *A* is the effective membrane area (m^2^), and ∆*P* is the operating pressure (Pa). To obtain the membrane pore size, the radius (*r*) was multiplied with 2.

#### 2.5.3. Membrane Wettability

The membrane surface hydrophilicity was estimated by measuring the contact angle of pristine PES membrane and MMM surface using a goniometer (Rame-hart 260, Succasunna, NJ, USA) [28]. A deionized water droplet was dropped (8 µL) on the membrane surface through the needle tip of the micro syringe at room temperature. The average value from 5 contact angle measurements at different locations captured by the magnified image of a camera was reported in this research work.

#### 2.5.4. Membrane Spectral Analysis

The functional groups of the samples were analyzed with Fourier transform infrared (FTIR) spectroscopy (Perkin Elmer, Waltham, MA, USA) from 500 to 4000 cm^−1^.

#### 2.5.5. HMO Nanoparticle Diffraction Analysis

An X-ray diffractometer (XRD) (X’Pert3Powder and Empyrean, PANalytical, Malvern, UK) was used to measure the X-ray diffraction patterns of the self-synthesized HMO nanoparticles using Cu Kα radiation at diffraction 2θ range 10° to 90° with a step size of 0.01° and exposure time of 60 s/step and 2°/step [29].

## 3. Results

### 3.1. Membrane Morphological Analysis

**Figure 2:**
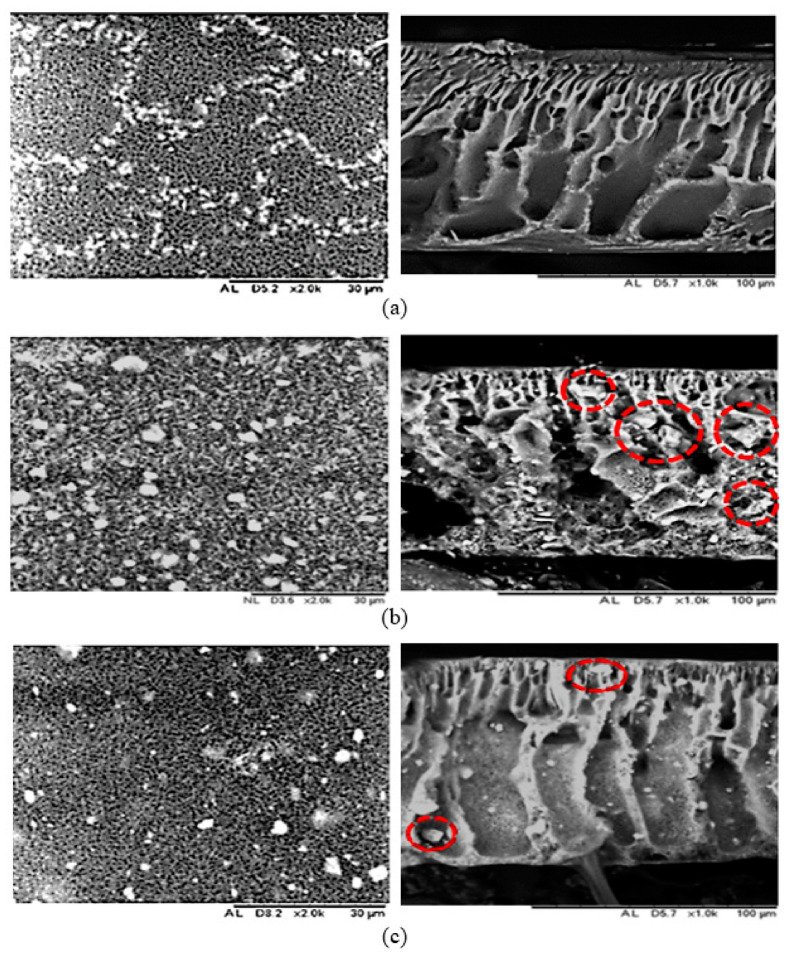

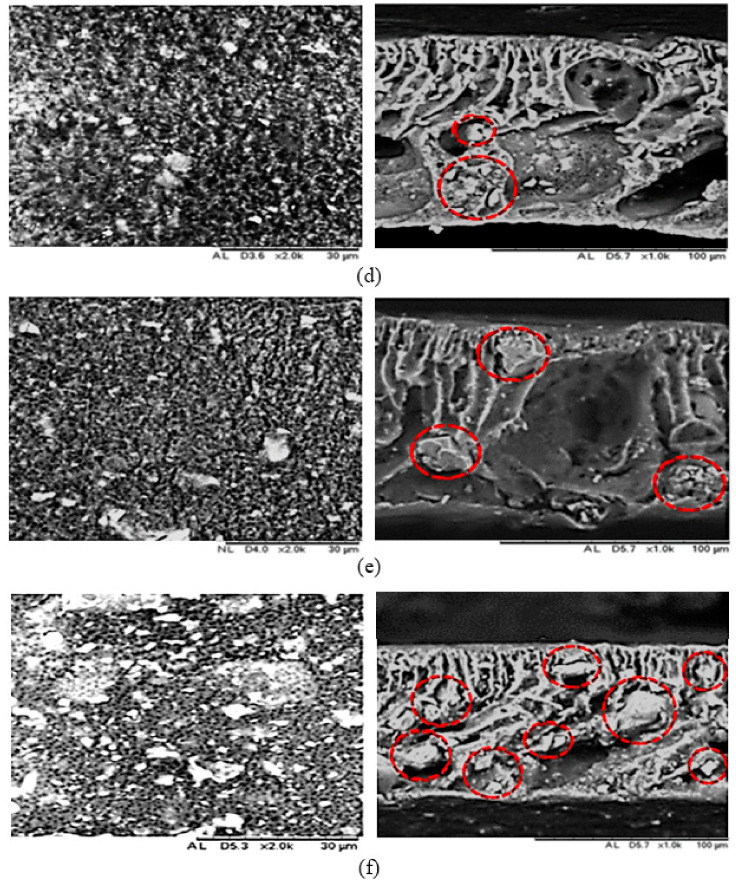
SEM images of top surface (left) and membrane cross-sectional morphology (right), (**a**) PES 0, (**b**) PES 1 and (**c**) PES 2, (**d**) PES 3, (**e**) PES 4, (**f**) PES 5. Red circles denote the aggregate of nanoparticles.

Figure 2 shows the surface and cross-sectional morphology of pristine PES and HMO/Al_2_O_3_-filled MMMs. The synergistic effects were clearly indicated in all the binary filler MMMs (PES 2, PES 3, and PES 4) cross-sectional images. According to Rosnan and her co-workers, the formation of finger-like structure was related to the viscosity of the dope solution, where high viscosity dope solution restrained the solvent and non-solvent exchange in phase inversion process [30]. Consequently, the formation of macro-void was restricted, resulting in poor membrane pore structure formation. Nanoparticles that possessed higher surface energy tended to migrate towards lower surface energy region, resulting in agglomeration. Reports reviewed by Gohari et al. and Lai et al. indicated that membranes that incorporated pure HMO nanoparticles in PES polymer matrix showed relatively high viscosity at 1118 cP and 570.1 mPa·s, respectively [10,27]. A similar trend was observed for PES 1 in this research work, which showed the formation of uneven finger-like structure, thus being in good agreement with the results reported by Lai et al. [10]. Although uneven membrane structure formed on PES 1, the membrane pore size improved significantly. The synergistic effect took place upon the introduction of binary fillers by evaluating the broader and wider membrane pore structure of PES 2 to PES 4. Shrinkage of the membrane film occurred as a result of the release of interfacial tension, forming larger macropores [14]. 

In this research work, agglomeration occurred in all the MMMs, and it was especially severe in PES 1 and PES 5. Among the MMMs, minimum agglomeration was observed in PES 3. Owing to the high Mn and Al content in PES 3, it promoted homogeneous dispersion of the nanofillers. This led to the release of interfacial stress, promotion of the migration of the binary fillers, and improvement in the membrane pore structure. With that, PES 3 achieved the highest mean pore size at 169 nm. In view of the SEM cross-sectional images in Figure 2, the incorporation of binary fillers into the polymeric matrix induced a positive synergistic effect that successfully mitigated agglomeration. Other than that, the thermodynamic and kinetic effects had close affinity with the membrane morphology, as described by Pang et al. [14]. From the thermodynamics point of view, the formation of the asymmetric membrane structure was due to the continuous de-mixing process through liquid–liquid phase separation, resulting in the exchange between the NMP solvent and water. The liquid phases in the ternary diagram consisted of polymer-rich and polymer-lean regions. Instantaneously, polymer precipitation occurred, resulting in polymer rearrangement in the polymer-rich phase until reaching the concentrated phase, and the pore formation occurred in the polymer lean phase. The formation of asymmetric membrane structure for PES 1 to PES 5 implied that an instantaneous de-mixing process occurred, which was closely related to the high polymer precipitation. These were in good agreement with the explanations provided by Arzhandi et al. [31].

Next, the formation of finger-like structure could be related to the hydrophilicity of the nanoparticles. HMO nanoparticles, which exist as aggregate in nature, tend to increase the dope solution viscosity. This was observed in the uneven membrane structure in PES 1, due to suppression of NMP–water exchange rate, restricting the water inflow. Consequently, thinner film of PES 1 was obtained. Incorporating Al_2_O_3_ nanoparticles reduced the dope solution viscosity, thus enhancing the NMP–water exchange rate because of the synergistic effect. The synergistic effect could be related to the release of interfacial stress, which caused the migration of nanoparticles during the phase inversion process, eventually increasing the membrane porosity [32]. Furthermore, the steric effects induced by the alumina nanofillers on the hydrophilic HMO nanofiller resulted in the formation of macropores among the binary filler MMMs (PES2 to PES4). This steric effect reaction was expressed in Reaction 1 [20].

(R1) $$Al-O-Al(s)+H_{2}O\left(g\right)↔Al-OH\left(s\right)+Al-OH\left(s\right)$$

On the basis of the surface morphology, the white color regions (agglomeration) were observed on the membrane pores from PES 1 to PES 5. This was probably due to the migration of nanoparticles from the higher surface energy to lower surface energy region, forming aggregates. In addition, the formation of macropore structure was likened as the margo structure in xylem, coupled with the minor aggregation of nanofillers, which was analogous with the torus structure in xylem.

### 3.2. Porosity and Pore Size Analysis

**Figure 3:**
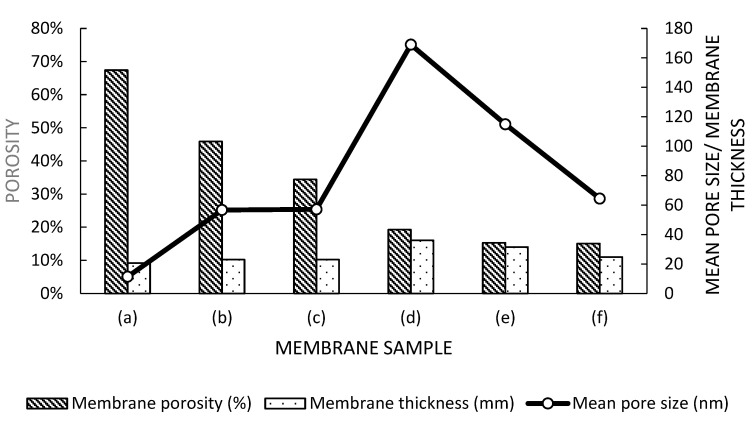
Comparison of porosity, thickness, and pore size of (**a**) PES 0, (**b**) PES 1, (**c**) PES 2, (**d**) PES 3, (**e**) PES 4, and (**f**) PES 5 membranes.

Interestingly, the thermodynamic stability and kinetic effects can be quantified through pore size and porosity of the membrane. Incorporating Al_2_O_3_ and HMO nanofillers enhanced the phase separation, thereby affecting the dope solution thermodynamic instability, resulting in the enhancement of phase separation rate and macropore stacking. The polymer from dope solution was readily precipitated; however, the hydrophilic properties of the nanoparticles with high interfacial energy caused them to leach out, resulting in formation of large pores in the membrane. Figure 3 shows the membrane thickness, porosity, and pore size of pristine PES membrane, pure HMO/PES MMMs, and HMO/Al_2_O_3_/PES MMMs at varying ratios and pure Al_2_O_3_/PES MMMs. 

It was observed that nanofiller-filled MMMs possessed membrane thickness of more than 10% compared with the pristine membrane. Upon introducing binary fillers, the membrane thickness increased by 3.6% compared with single-filler MMMs. This could be explained by the thermostability of the dope solution based on ternary diagram of PES/NMP/water system. Initial observation of gelatin indicated the cloud point of the binodal curve. According to Arzhandi et al., incorporating nanoparticles into the membrane would cause the shift of the bimodal curve towards the solvent axis due to the thermodynamic stability distortion, allowing phase inversion at low water concentration [30]. Hence, the addition of hydrophilic nanoparticles would expedite the system entering the immiscibility gap. Eventually, less water was required for polymer precipitation [33]. As a result, instantaneous de-mixing occurred upon reaching the binodal curve, inducing rapid NMP–water exchange. Consequently, the formation of a thicker membrane occurred [14]. Meanwhile, mean pore size showed an increasing trend from PES 0 to PES 3 and reduced from PES 3 to PES 5. In contrast, membrane porosity decreased upon the addition of nanofillers and reduced HMO nanoparticles’ concentration in binary filler membrane [28]. This may have been due to the nanofillers having occupied the membrane pore wall structure. According to Nasrollahi et al. and Xia et al., formation of larger membrane pore structure were due to the migration of the nanoparticles towards the polymer surface [34,35]. In this research work, 0.5:0.5 ratio HMO/Al_2_O_3_ (*w*/*w*) nanoparticles used in PES 3 were determined as the optimal ratio. Further decreasing the HMO weight percentage and increasing in the A_2_O_3_ weight percentage would affect the polymer precipitation process, affect the mean pore size, and eventually affect the membrane flux performance. 

From Figure 3, it was observed that membrane mean pore size increased by more than 400% upon incorporation of fillers. A 196% increase in mean pore size from PES 2 to PES 3 MMMs was obtained upon optimizing the HMO and Al_2_O_3_ ratio through reduction of HMO nanoparticle weight percentage and increase in Al_2_O_3_ weight percentage. Beyond this optimal ratio, the mean pore size decreased.

### 3.3. Wettability Analysis

Wettability study was conducted to determine the hydrophilicity of the membrane by evaluating the water contact angle. The addition of highly hydrophilic HMO/Al_2_O_3_ nanoparticles into the PES polymeric membrane would increase the substrate–vapor surface tension [36]. Once the substrate–vapor surface tension exceeds the solid–liquid surface tension, the water droplets would be dragged towards the higher surface tension region, hence reducing the liquid–vapor surface tension. As a result, reduction of water contact angle would be obtained. Figure S1 shows the water contact angle formed by the hydrophilic MMMs. *ϓ_SG_*, *ϓ_LG_*, and *ϓ_SL_* denote substrate–vapor surface tension, liquid–vapor surface tension, and substrate–liquid surface tension, respectively.

**Figure 4:**
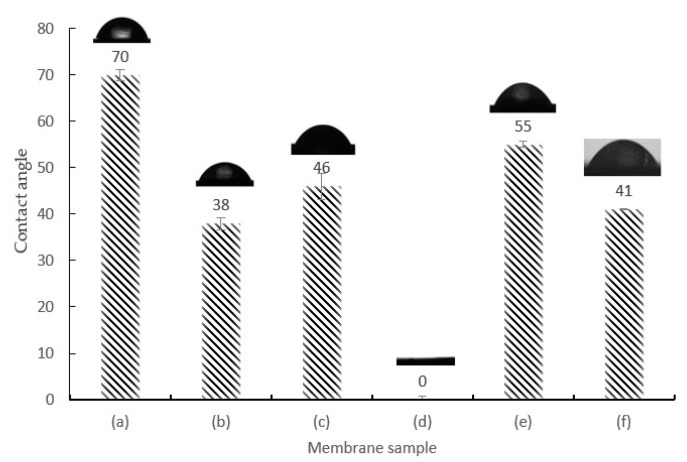
Wettability results of (**a**) PES 0, (**b**) PES 1, (**c**) PES 2, (**d**) PES 3, (**e**) PES 4, and (**f**) PES 5 membranes.

The wettability study analysis from Figure 4 revealed PES 3 MMMs to be superhydrophilic, wherein the water contact angle achieved 0°. Compared to the pristine PES membrane’s water contact angle at 70°, incorporation of HMO nanoparticles in PES 1 significantly reduced the water contact angle by 45% to 38°, which proved the success of introducing highly hydrophilic properties on the membrane surface [14,17]. However, a slight increase in water contact angle to 46° upon the reduction of HMO nanoparticle weight percentage was observed. A high wettability effect on PES 3 was a clear indication of the synergistic effect of HMO nanoparticles and Al_2_O_3_ in the polymeric matrix. The high wettability effect of PES 3 was further supported by the high pure water flux, which achieved 67% of flux efficiency increment compared to PES 1, which used single HMO filler. There was a significant decrease in water contact angle from PES 2 to PES 3, which may have been due to the higher Mn and Al content. This may result in increased mean pore size and membranes’ hydrophilicity, especially for PES 3. With the abundant membrane pores with large mean pore size, a hydration layer was formed on the membrane pore structure, aided by capillary action in achieving continuous water penetration across the membrane. This caused PES 3 membrane to achieve high hydrophilicity (i.e., superhydrophilic). Further reducing the HMO nanoparticles in PES 4 and PES 5 MMMs caused the membrane to decrease in hydrophilicity. Consequently, the water contact angle in PES 4 and PES 5 increased up to 55° and 41°, respectively. In this research work, the overall water contact angle from the wettability study showed a normal distribution trend by considering the pristine PES 0, pure HMO PES 1, binary filler PES 3, and pure Al_2_O_3_ PES 5 MMMs. 

### 3.4. Spectral Analysis

**Figure 5:**
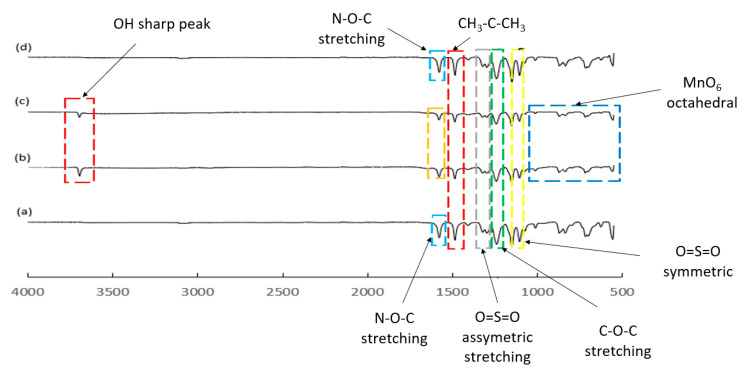
FTIR spectra of (**a**) PES 0, (**b**) PES 1, (**c**) PES 3, and (**d**) PES 5 membranes.

To confirm the incorporation of HMO nanoparticles into the PES polymer matrix, FTIR spectral analysis was performed. The analysis of membrane surface functional group is summarized in Figure 5.

On the basis of Figure 5, the peaks between 3680 cm^−1^ and 3710 cm^−1^ for PES 1 and PES 3 indicated the presence of -OH in water. HMO was hydrophilic in nature, and hence the presence of these peaks implied successful incorporation of HMO nanoparticles into the membrane. There were no obvious -OH peaks observed from the pristine PES membrane and Al_2_O_3_-nanofiller-incorporated PES MMMs. Comparing between HMO and Al_2_O_3_ nanofillers’ hydrophilicity, it was found that HMO-incorporated membranes showed higher hydrophilicity than Al_2_O_3_ membranes. The reduction in intensity of the -OH peak from PES 1 to PES 3 supported this theory. Next, the O-H bending vibration in the spectra range from 1619 to 1679 cm^−1^ showed the presence of Mn-O, indicating the successful incorporation of HMO nanoparticles in the PES membrane. The bending was especially obvious in PES 1 compared to PES 5 due to the strong hydrophilic properties of HMO nanoparticles. However, the intensity of bending gradually decreased upon the reduction of HMO nanoparticles, due to the reduction of O elements. In addition, the weak bands occurring within the spectra range of 400 to 900 cm^−1^ pertained to MnO_6_ octahedral, which was present in the self-synthesized HMO nanoparticles [26,36]. The presence of N-O-C functional group at approximately 1600 cm^−1^ showed the presence of NMP solvent in PES polymer matrix. Next, the peak at 1322 cm^−1^, 1298 cm^−1^, 1151 cm^−1^, and 1153 cm^−1^ in PES 0, PES 1, PES 3, and PES 5, respectively, indicated the presence of strong sulfone group as the backbones of PES. The stretching vibration at 1240 to 1242 cm^−1^ of C-O-C bond resulted from the weak absorption of O element from the ether group, as described by Gohari et al. [27]. Furthermore, the presence of O=S=O symmetric and asymmetric stretching at around 1500 and 1100 cm^−1^ indicated the presence of sulfone group. Lastly, the functional group at 1487 cm^−1^ indicated the presence of CH_3_-C-CH_3_ stretching, as described by Lai et al. [10]. 

On the basis of the FTIR spectra analysis, it was found that PES 0 and PES 5 did not display significant difference in OH stretching compared to the HMO nanoparticle filled MMMs. This indicated that Al_2_O_3_ was less hydrophilic compared to HMO nanoparticles. Furthermore, the limitation of FTIR spectrum in identifying the chemical elements required an alternative characterization in determining the presence of Al_2_O_3_ in MMMs. According to the research work conducted by Lai et al., -OH broad peak was observed among TiO_2_ MMMs and pristine PES with the incorporation of PVP pore opening agent. However, there was only minor difference of the broad peak in -OH stretching range at 3200 to 3700 cm^−1^, which could have been due to the effect of PVP [10]. In this research work, no pore opening agent was used. In the meantime, the presence of -OH bending on the hydrocarbon group (CH_3_-C-CH_3_), sulfone group, and C-O-C in PES 1 and PES 3 indicated the presence of HMO nanoparticles in the PES matrix.

### 3.5. Energy Dispersion X-ray (EDX) Analysis

EDX analysis was used to identify the elements weight percentage in the membrane samples. Table 1 shows the elemental analysis of the pristine PES membrane and MMMs. 

From Table 1, it was observed that pristine PES membrane had the lowest O content compared to other MMMs. Upon adding HMO filler for PES 1, the O element increased by 69.7%. After the addition of the optimum ratio of binary filler at PES 3, the O element further increased by almost 2% compared to PES 1. Further reducing HMO nanofillers and increasing Al_2_O_3_ nanofiller loading in PES 4 and 5 reduced O content. From the water contact angle study, there was a sharp decline in water contact angle from PES 2 to PES 3, which indicated that PES 3 membrane’s hydrophilicity improved significantly. An important discovery from the EDX analysis showed that the Al and Mn contents were higher in PES 3, PES 4, and PES 5. A total of 15.28% of Al element in PES 5 ascertained the incorporation of Al_2_O_3_ into the PES polymer matrix. This indicates that the nanofillers incorporated in PES 3, PES 4, and PES 5 were much higher than PES 1 and PES 2. For PES 3, the Mn content was the highest among all samples. This indicates that PES 3 had better HMO nanoparticles incorporation. This led to PES 3 achieving good membrane separation performance and anti-fouling properties. 

### 3.6. XRD Diffraction Analysis

**Figure 6:**
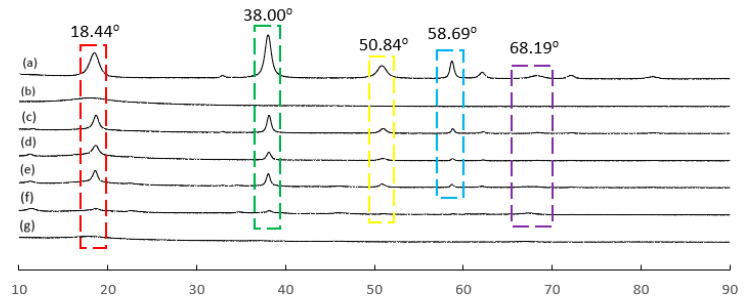
X-ray diffraction pattern of (**a**) HMO nanoparticles, (**b**) PES 0, (**c**) PES 1, (**d**) PES 2, (**e**) PES 3, (**f**) PES 4, and (**g**) PES 5.

X-ray diffraction analysis was also performed to further confirm the incorporation of HMO nanoparticles into the PES polymeric matrix. Figure 6 shows the X-ray diffraction pattern of HMO nanoparticles, PES 0, PES 1, PES 2, PES 3, PES 4, and PES 5 membranes. The highlighted regions show the peaks obtained from the analysis.

The peaks at 18.44°, 38.00°, and 68.19° were in line with the results obtained by Gohari et al. and Al-Husaini et al., which showed the successful incorporation of HMO nanoparticles in the membrane [26,27]. The sharp peaks observed at 50.84° and 58.69° were probably induced by the hydroxyl group [26]. Hence, it indicated that HMO particles were highly hydrophilic as the reduction of HMO nanoparticles tends to diminish the intensity of the -OH peak, which was in good agreement with the FTIR result. In addition, no significant peak was observed in PES 5, except a minor peak at 68.19°, which justified the presence of Al_2_O_3_ in the PES polymeric matrix.

### 3.7. Membrane Separation Performance

**Figure 7:**
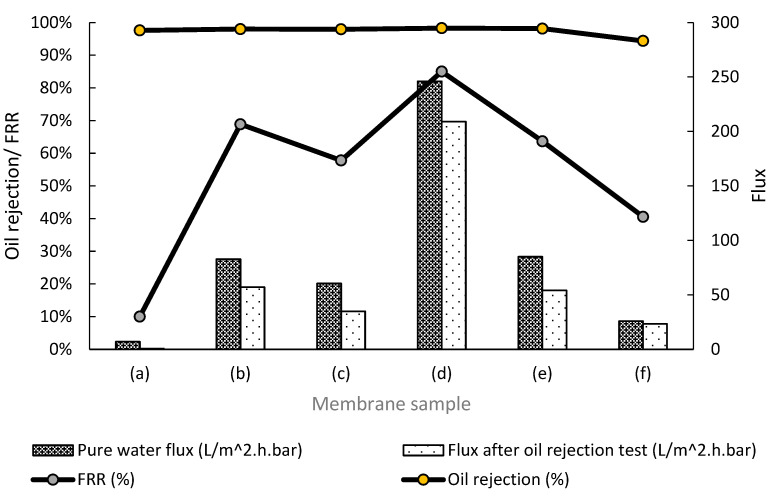
Pure water flux, water flux after oil rejection and flux recovery ratio of (**a**) PES 0, (**b**) PES 1, (**c**) PES 2, (**d**) PES 3, (**e**) PES 4, and (**f**) PES 5.

The efficiency of membrane separation for oily wastewater treatment relied on the initial water flux, flux after oil rejection test, flux recovery ratio, and oil rejection. High oil concentration (12 g/L or 12,000 ppm) was used in this research work to evaluate the membrane separation performance. Hence, the membrane performances for PES 0 to PES 5 are summarized in Figure 7.

On the basis of Figure 7, the pure water flux, flux after oil rejection test, and flux recovery ratio percentage of PES 0 were 270.0%, 3218.6%, and 305.4% lower compared to other MMMs, respectively. The low flux performance of PES 0 was probably due to small membrane mean pore size, as no hydrophilic pore opening agent was added. Many research works showed good performance of organic-based hydrophilic pore opening agent, such as PVP [37,38]. However, addition of PVP into the dope solution tends to increase the viscosity of the dope solution, causing inhomogeneity in distribution of the nanoparticles in the dope solution. Therefore, exceptionally high operating pressure on pristine PES membrane was required to obtain a small flux value of 0.7 L/m^2^.h.bar after oil rejection test. According to Huang et al., high operating pressure exerted on the oil droplets would form a continuous oil film, causing coalescence of the oil particles in membrane pores [39]. With that, the pristine membrane experienced significant fouling, incurring poor flux after oil rejection test and flux recovery ratio percentage.

Upon adding HMO nanofillers in PES 1, pure water flux, flux after oil rejection test, flux recovery ratio percentage, and oil rejection percentage improved by 1088.0%, 8045.7%, 588.7%, and 0.4%, respectively. According to Figure 3, the mean pore size of PES 1 improved by 398.3%, providing a larger path for water to pass through the membrane, without compromising the oil rejection percentage. Comparing PES 3 with PES 1, the pure water flux, flux after oil rejection test, flux recovery ratio percentage, and oil rejection percentage for PES 3 improved by 197.1%, 266.6%, 23.4%, and 0.3%, respectively. The significant improvement on membrane separation performance and anti-fouling properties of PES 3 was attributed to its large mean pore size. Water was trapped in the membrane pore structure, generating a hydration layer and creating a repulsive barrier for oil particles to prevent membrane fouling. Moreover, the capillary action on the xylem-like membrane pore structure further enhanced the movement of the water molecule, improving the water flux [40].

Further reduction of HMO nanofiller and increasing Al_2_O_3_ loading reduced the membrane’s mean pore size, which affected the membrane separation performance. Declination of mean pore size resulted in accumulation of oil molecules on membrane surface, formed a cake layer, and caused water restriction into membrane pores [41]. It was observed that PES 1 showed better separation performance than PES 5, where its pure water flux, flux after oil rejection, flux recovery ratio, and oil rejection percentage were higher by 68.9%, 59.3%, 41.1%, and 3.7%, respectively. A directly proportional relationship was observed on the basis of the mean pore size from Figure 3 and membrane separation performance from Figure 7 with a normal distribution curve.

**Figure 8:**
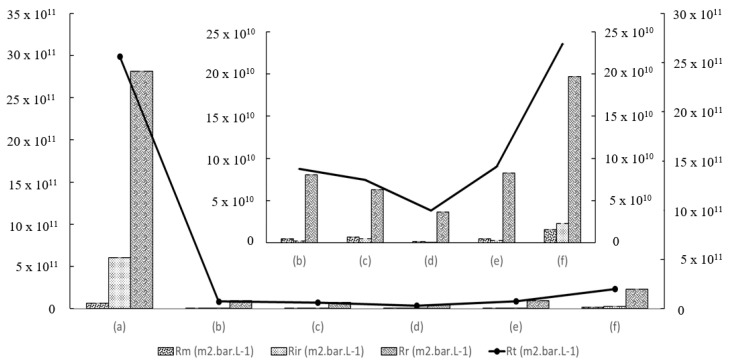
Profile of reversible, irreversible, and total resistances for (**a**) PES 0, (**b**) PES 1, (**c**) PES 2, (**d**) PES 3, (**e**) PES 4, and (**f**) PES 5. Inset represents the magnified graph of (**b**) PES 1, (**c**) PES 2, (**d**) PES 3, (**e**) PES 4, and (**f**) PES 5.

In addition, the membrane separation performance was corroborated with the results from membrane fouling tests. On the basis of the results shown in Figure 8, it was found that the total resistance for PES 0 was at least 93% higher than other MMMs. Once the HMO nanofillers were added into the PES polymer matrix, the total membrane resistance improved by 97.0%. PES 3 possessed the lowest membrane total resistance, owing to its largest membrane mean pore size, leading to less oil deposition on the membrane pore structure. On the other hand, PES 5 incurred 145% higher total resistance than PES 1. This might have been due to lower hydrophilicity of the membrane surface, smaller mean pore size, and lower porosity, which caused aggregation of oil molecules on the membrane surface. Moreover, small mean pore size and porosity also severely affected the membrane resistance. 

The separation performance of the as-synthesized membranes was compared with various additive blended PES membranes by other researchers. Table 2 summarizes their water contact angle and membrane performance for oily wastewater treatment. 

In this work, the membrane separation performance was slightly higher compared to the binary fillers MMMs of Lai et al. [10]. The operating condition of this work was almost similar to that conducted by Gohari et al. and Lai et al. [10,27]. The overall flux for PES 1 to PES 5 membranes obtained in this work was 50% to 400% higher than the performance reported by Lai et al. [10], indicating a positive contribution of this research. 

## 4. Conclusions

In this work, a new superhydrophilic HMO-Al_2_O_3_-modified PES mixed matrix membrane was developed for oily wastewater treatment. Although pore opening agent was not used during membrane fabrication, the nanofiller-incorporated membranes showed superior antifouling and membrane separation performance, owing to the highly hydrophilic HMO and Al_2_O_3_ nanoparticles. PES 3 with the largest membrane mean pore size at 169 nm achieved the highest pure water flux (245.95 L/m^2^.h.bar) and water flux after oil rejection (209.06 L/m^2^.h.bar), with 85% flux recovery ratio, without compromising the oil rejection performance of 98.27%. Moreover, PES 3 achieved significant water contact angle improvement, which was close to 0^o^, proving its strong hydrophilicity. Its superior performance and antifouling properties were contributed by the formation of hydration layer, inducing extraordinary repulsive barrier, hence resulting in strong resistance to oil droplet fouling. Although the hydrophilic HMO/Al_2_O_3_ nanofillers were able to develop a highly hydrophilic membrane, excessive loading resulted in severe nanoparticles aggregation, ultimately deteriorating the membrane separation performance. Finally, HMO and Al_2_O_3_ loading was optimized at 0.5:0.5 wt % to achieve significant membrane separation performance without compromising foulant rejection performance in high-concentration oily wastewater treatment application.

## Figures and Tables

**Table 1:** EDX analysis for PES 0 to PES 5.

Membrane	Element (wt %)				
	C	O	S	Al	Mn
PES 0	62.70	22.93	14.37	0.00	0.00
PES 1	52.59	38.91	7.89	0.00	0.61
PES 2	50.21	39.33	8.76	0.97	0.73
PES 3	45.22	39.68	8.82	5.35	0.94
PES 4	46.14	38.46	8.65	6.47	0.27
PES 5	51.43	25.32	7.97	15.28	0.00

**Table 2:** Summary of the performance of additives in PES membranes for oily wastewater treatment application.

Additives	Filler Loading	Water Contact Angle	Membrane Performance *	Ref.
PVP/HMO	10.00%	58.7°	PWF:210; R:93	[42]
PVP/HMO	23.08%	16.4°	PWF:573.2; R:94; FRR:75	[27]
PVP/HMO/TiO_2_	23.08%	<10°	PWF:29; R:99; FRR:91.5	[10]
HMO/Al_2_O_3_	23.35%	$\approx 0^{o}$	PWF:245.95; R:98.27; FRR:85	This work

* PWF: pure water flux (L/m^2^.h); R: rejection (%); FRR: flux recovery ratio (%).

## Data Availability

Not applicable.

## Supplementary Materials

The following supporting information can be downloaded at: https://www.mdpi.com/article/10.3390/membranes12090860/s1, Figure S1: Water contact angle formed by the hydrophilic PES/HMO/Al_2_O_3_ MMMs; Figure S2: Water contact angle plot and angle value.; Table S1: Membrane thickness, porosity and mean pore size.
