# Supplementary material for: Xylem-Inspired Hydrous Manganese Dioxide/Aluminum Oxide/Polyethersulfone Mixed Matrix Membrane for Oily Wastewater Treatment
Source: Membranes (Basel). 2022 Sep 5;12(9):860. doi: 10.3390/membranes12090860 (PMC9501045; doi:10.3390/membranes12090860)
Supplement: Supplementary file 1 [file membranes-12-00860-s001.zip › membranes-1826859-supplementary.pdf]

Supplementary Information

Table S1. Thickness of membrane, membrane porosity and mean pore size.

Membrane	Membrane thickness (mm)	Membrane porosity (%)	Mean pore size (nm)
PES-0	0.092	67.38%	11.37
PES-1	0.102	45.85%	56.66
PES-2	0.102	34.39%	57.12
PES-3	0.116	19.26%	169.00
PES-4	0.114	15.28%	114.87
PES-5	0.110	15.06%	64.41

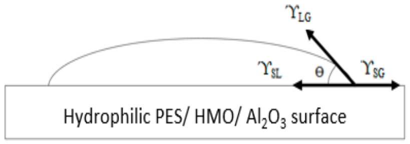

Figure S1. Water contact angle formed by the hydrophilic PES/HMO/Al_2O_3 MMMs.

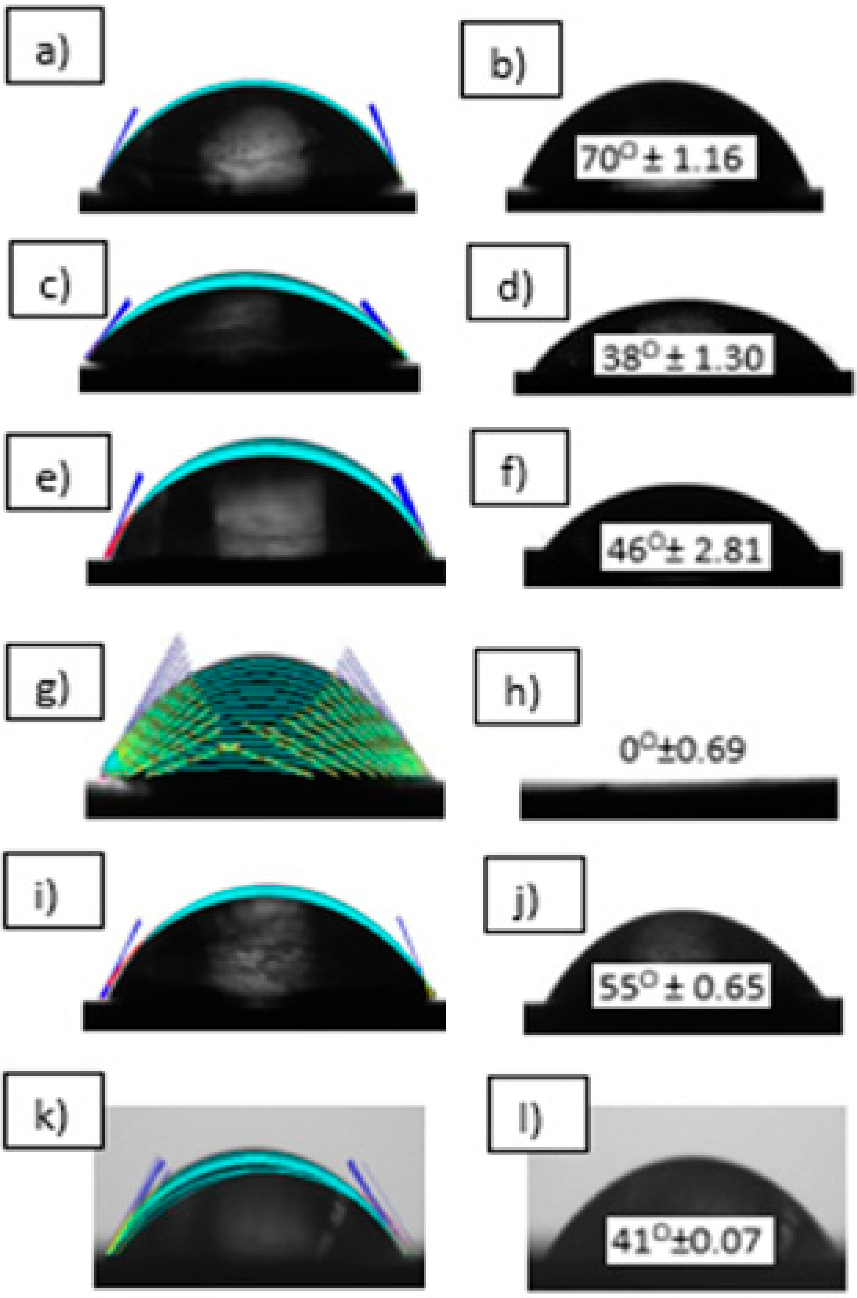

Figure S2. Water contact angle (WCA) of (a, b) PES 0, (c, d) PES 1, (e, f) PES 2, (g, h) PES 3, (i, j) PES 4 and (k, l) PES 5. Left images indicate water contact angle plot, while the right images indicate water contact angle values.
